# Advances in the use of microsurgery in neurogenic tumors: a bibliometric and visualization analysis

**DOI:** 10.1097/JS9.0000000000001899

**Published:** 2024-07-02

**Authors:** Zixin Luo, Xinyue Song, Duoqin Huang, Kang Zou, Li Xiao

**Affiliations:** a The First Clinical Medical College, Gannan Medical University; b Department of Critical Care Medicine, The First Affiliated Hospital of Gannan Medical University; c Department of Rehabilitation Medicine, The First Affiliated Hospital of Gannan Medical University, Ganzhou City, Jiangxi Province, People’s Republic of China

Highlights

- This study is the first bibliometric analysis of studies on the use of microsurgery in neurogenic tumors.
- The United States is the leading country in research on the use of microsurgery in neurogenic tumors.
- Thematic analysis reveals the use of imaging techniques in glioma resection as a potential research hotspot.

*Dear Editor,*

In recent years, with the continuous improvement of microsurgical techniques and the development of neuroimaging, more and more studies have shown that the application of microsurgery in the treatment of neurogenic tumors is remarkable, and microsurgery has become one of the preferred methods for the treatment of neurogenic tumors^1^. Through microsurgery, surgeons can clearly observe the boundaries of the tumor and the structure of the surrounding nerve tissue to locate and remove the tumor more accurately, reduce nerve damage and intraoperative bleeding, and improve the success rate of the surgery and the quality of the patient’s life^2^.

To ascertain the global research trends in microsurgery for neurogenic tumors, we employed bibliometric analysis utilizing R-bibliometrix, VOSviewer 1.6.19, and CiteSpace 6.2.R4. Relevant literature was obtained from the Web of Science Core Collection on 10 March 2024 for searching. We have set the time span from 1 January 1988 to 31 December 2023, and conducted searches using the keywords “pituitary adenoma,” “neuroblastoma,” or “neurofibroma,” along with “microsurgery” or “microsurgery surgery.” We limited the publication types to reviews and original articles and excluded non-English literature, ultimately including 428 articles in our analysis. Figure 1A shows the quantity of annual publications and the quantity of annual citations related to this topic. There was an overall steady increase from 1988 to 2023 despite fluctuations in the number of relevant publications, and the overall range of fluctuations in the number of annual citations was large. Of the 428 articles included, a total of 1037 institutions and representatives from 151 countries were involved. Figure 1B illustrates a world map of the co-authorship network composed of 26 countries, with the majority being European nations, and the size of the nodes indicates the number of papers associated with each node. The United States of America (USA) leads the world in the number of publications (*n*=84), with China ranking second (*n*=39), followed by Germany (*n*=18). Figure 1C constructs the institution co-occurrence graph using CiteSpace. Where each node represents an institution, the size of the node represents the number of times the institution has been cited, and the connections between the nodes represent the collaborative relationships between institutions. Notably, the most cited institution was the Mayo Clinic (TC=1398), followed by the University of Virginia (TC=860).

**Figure 1:**
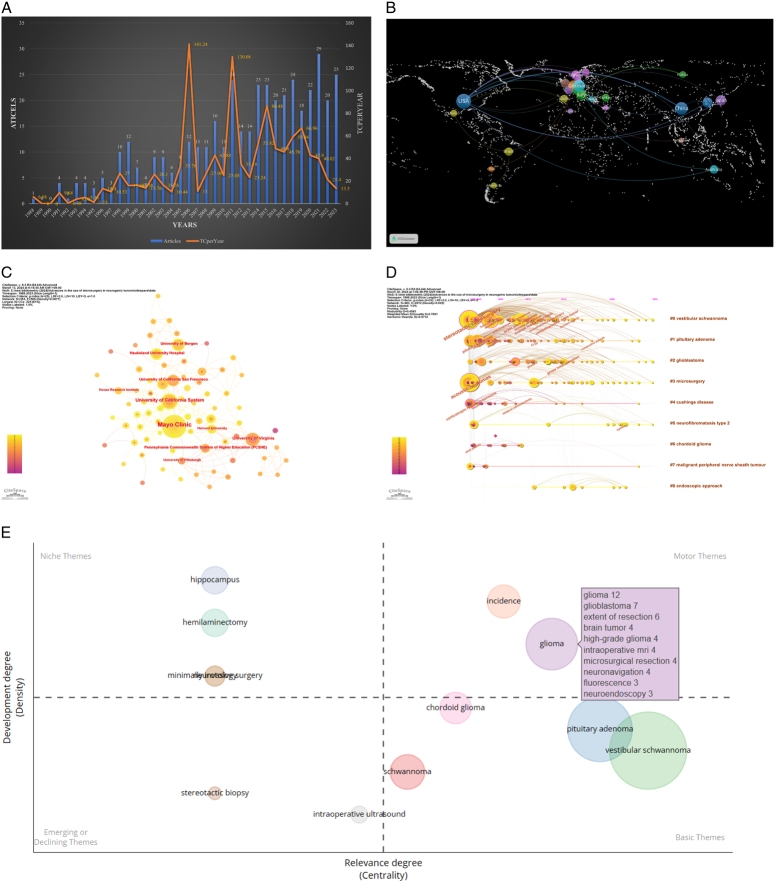
(A) Annual output of research on the use of microsurgery in neurogenic tumors. (B) World map of cooperation networks among 26 countries. (C) Institutional co-occurrence analysis charts in CiteSpace. (D) Timeline visualization of keyword. (E) Timeline graph of the top 9 keywords.

CiteSpace created a timeline graph of the keywords to observe the evolution of the keywords, and it can be observed that the earlier more active clusters are #0 vestibular schwannoma, #3 microsurgery, and #1 pituitary adenoma, while the more recent more active clusters are #2 glioblastoma and #5 neurofibromatosis type2 (Fig. 1D). In Figure 1E Thematic Maps, the horizontal coordinate represents mediating centrality, which represents the degree of relevance of the theme to the field, and the vertical coordinate represents density, which indicates the degree to which the theme has developed in the field. Quadrant 1 (top right) represents Motor themes, which are both important and well-developed; quadrant 2 (top left) represents Highly Developed and Individual themes that are well-developed but not important to the current domain; quadrant 3 (bottom left) Emerging or Declining Themes. The third quadrant (bottom left) represents emerging or declining themes, which are also not well-developed and may be emerging or disappearing, while the fourth quadrant (bottom right) represents basic and transversal themes that are important to the field but are not well-developed.

Through Bibliometrix’s thematic map analysis (Fig. 1E), we shifted our research focus to the first quadrant, focusing on microsurgical imaging techniques in gliomas such as intraoperative MRI, neuronavigation, and fluorescence. Glioma is an aggressive CNS tumor that penetrates into the surrounding brain tissue. This feature complicates the treatment of endogenous brain tumors, both low-grade (LGG, WHO grade II) and high-grade gliomas (HGG, WHO grades III and IV)^3^. A growing number of studies have shown that the extent of surgical resection is an important prognostic factor for progression-free survival (PFS) and overall survival (OS)^4–7^. The complexity of tumor heterogeneity and the existence of cells located beyond the contrast-enhanced areas identified in preoperative MRI scans present significant challenges in accurately delineating tumor boundaries through both preoperative imaging modalities and intraoperative assessments^3^. Tools such as intraoperative MRI (iMRI), neuronavigation, intraoperative ultrasonography (IOUS), and intraoperative imaging techniques are utilized during glioma resection procedures to enhance the precision of tumor removal and ultimately enhance patient outcomes^8^.

The application of microsurgery in neurogenic tumors is indeed a complex and challenging field. The tumors are often hidden and closely related to surrounding neural structures, requiring meticulous surgical care to avoid damage. Their slow and inconspicuous growth can lead to misdiagnosis or missed diagnosis, complicating surgical treatment. Moreover, multidisciplinary collaboration is essential, involving neurosurgery, radiation therapy, chemotherapy, and other expertise. Despite these challenges, the unwavering efforts of medical professionals and teamwork can lead to improved patient outcomes and quality of life.

In summary, the bibliometric analysis of microsurgery for neurogenic tumors has uncovered significant research focal points and trends, highlighting the profound impact of integrating imaging technologies with minimally invasive surgical techniques on the future of precision medicine. Concurrently, it underscores the imperative to prudently evaluate the challenges and risks associated with the advancement of these transformative technologies to ensure the safety and quality of patient care.

## Ethical approval

Not applicable.

## Consent

Not applicable.

## Source of funding

This work was supported by the Ganzhou Science and Technology Bureau, China (GZ2018ZSF074); Ganzhou Science and Technology Bureau, China (2023LNS36644); and Jiangxi Provincial Health Commission, China (202410348).

## Author contribution

K.Z. and L.X.: conceptualization; Z.L.: writing – original draft, data curation, and formal analysis; K.Z.: funding acquisition, investigation, and methodology; L.X.: project administration, visualization, and writing – original draft; Z.L. and D.H.: resources, software, supervision, and validation; D.H.: writing – original draft; X.S.: methodology and project administration; X.S. and K.Z.: writing – review and editing. All authors contributed to the article and approved the final version for submission.

## Conflicts of interest disclosure

The authors declare no conflicts of interest.

## Research registration unique identifying number (UIN)

Not applicable.

## Guarantor

All authors.

## Data availability statement

Data are available from the corresponding author upon reasonable request and with the permission of the corresponding author.

## Provenance and peer review

Not applicable.
